# Time to bury the chisel: a continuous dorsal association tract system

**DOI:** 10.1007/s00429-024-02829-w

**Published:** 2024-07-16

**Authors:** Emiel van den Hoven, Marco Reisert, Mariacristina Musso, Volkmar Glauche, Michel Rijntjes, Cornelius Weiller

**Affiliations:** 1 Department of Neurology and Clinical Neuroscience, Medical Center, Faculty of Medicine, University of Freiburg, Breisacher Straße 64, 79104 Freiburg, Germany; 2 Department of Radiology – Medical Physics, Medical Center, Faculty of Medicine, University of Freiburg, Killianstraße 5a, Freiburg, 79106 Germany

**Keywords:** Arcuate Fasciculus, Tractography, Repetition, Aphasia

## Abstract

The arcuate fasciculus may be subdivided into a tract directly connecting frontal and temporal lobes and a pair of indirect subtracts in which the fronto-temporal connection is mediated by connections to the inferior parietal lobe. This tripartition has been advanced as an improvement over the centuries-old consensus that the lateral dorsal association fibers form a continuous system with no discernible discrete parts. Moreover, it has been used as the anatomical basis for functional hypotheses regarding linguistic abilities. Ex hypothesi, damage to the indirect subtracts leads to deficits in the repetition of multi-word sequences, whereas damage to the direct subtract leads to deficits in the immediate reproduction of single multisyllabic words. We argue that this partitioning of the dorsal association tract system enjoys no special anatomical status, and the search for the anatomical substrates of linguistic abilities should not be constrained by it. Instead, the merit of any postulated partitioning should primarily be judged on the basis of whether it enlightens or obfuscates our understanding of the behavior of patients in which individual subtracts are damaged.

**Supplementary Information:**

The online version contains supplementary material available at 10.1007/s00429-024-02829-w.

The arcuate fasciculus (AF) may be subdivided into a subtract directly connecting inferior frontal gyrus (IFG) (and possibly precentral gyrus, PreCG) and the posterior temporal lobe, and two indirect subtracts that form an indirect connection between IFG/PreCG and the posterior temporal lobe (Catani et al. 2005; Martino et al. 2013). Among the latter two subtracts, the anterior one connects IFG/PreCG to “Geschwind’s territory” (defined by Forkel et al. (2020) as temporo-parietal junction (TPJ) and posterior supramarginal gyrus (pSMG)). The posterior one (pArc) connects Geschwind’s territory to the posterior temporal lobe (Weiner et al. 2017). In a recent Brain Structure and Function article, Roelofs Ardi (2024), elaborating on discussion raised by Forkel et al. (2020), hypothesized that damage to the direct tract impedes the capacity to repeat single multisyllabic words, whereas damage to the indirect tracts impedes the capacity to repeat sequences of words. We point out two issues with Roelofs’ otherwise enlightening re-evaluation of Wernicke’s idea of the “psychological reflex arc”. First, we contest an interpretation of Wernicke by Roelofs regarding the role of the AF in word production. And second, we argue that the widely accepted anatomical tripartition presupposed by Roelofs’ hypothesis is potentially redundant.

## Wernicke on the white matter tracts necessary for word production

Roelofs (2024), citing Wernicke, claims that

[w]ord production involves the fiber tracts “that connect the frontal lobes to the occipito-temporal lobes in the white matter of the hemisphere, especially Burdach’s arched bundle” (p. 34), referring to the AF (...)

**Fig. 1:**
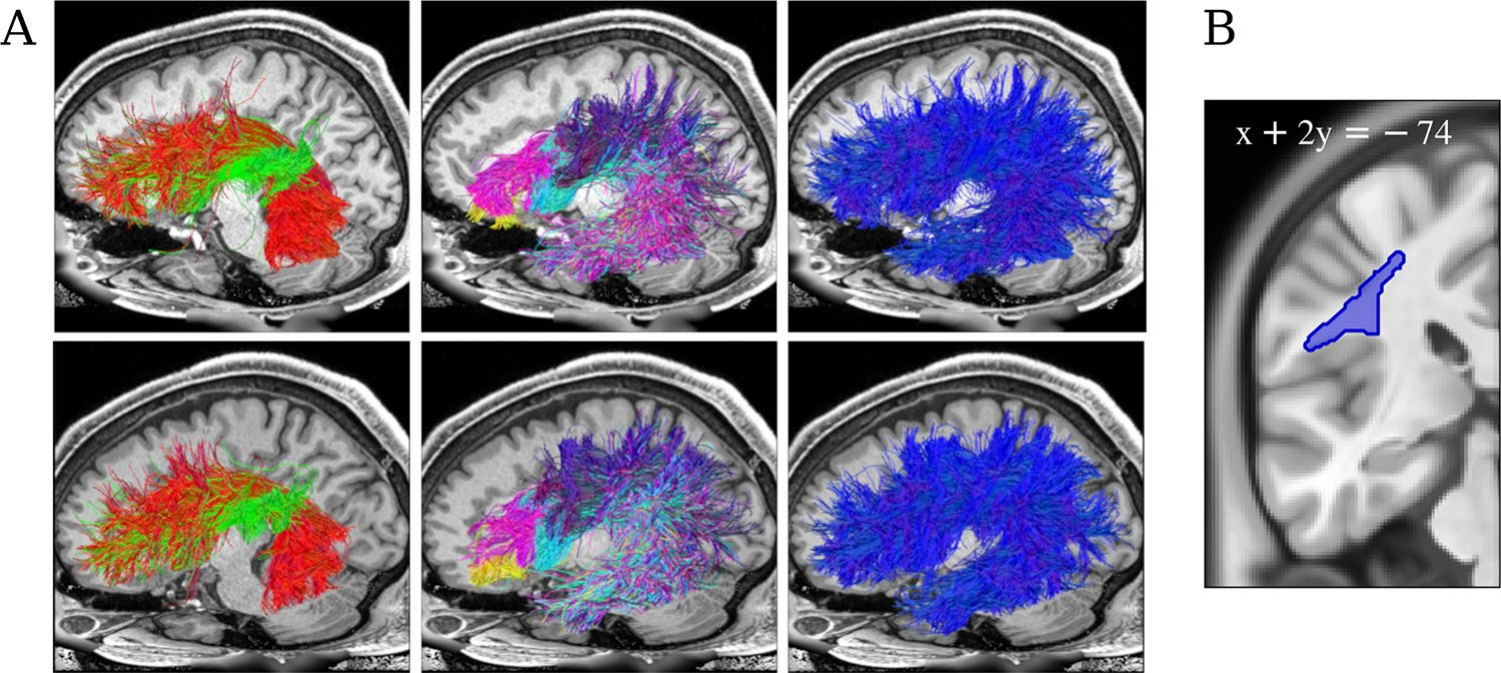
Subtracts of the dorsal stream. **A** Arcuate Fasciculus sub- and supertracts for two randomly selected HCP subjects (top: 125222, bottom: 133827) in their native spaces. Left: dorsal streamlines ending in posterior temporal lobe (red) and Geschwind’s territory (green). Center: dorsal streamlines ending in pars triangularis (magenta), pars opercularis (cyan) and pars orbitalis (yellow) of the IFG, and PreCG (purple). Right: The dorsal stream: all streamlines that pass through a dorsal white matter ROI at the central sulcus with an anterior-posterior orientation (i.e., $\left| \text {cos}(\theta _{\textbf{u}, \textbf{v}}) \right| > \tau$, where $\textbf{u}$ is the streamline tangent, $\textbf{v} = [0, 1, 0]$ and $\tau = 0.9$), except those which also traverse a 7 mm-radius spherical ROI located at the extreme capsule with the same orientation as ventral tract fibers (MNI coordinate: $(-32, 3, -10)$; $\textbf{v} = [1, 2, 1]$; $\tau =0.9$). **B** The dorsal white matter ROI in MNI space

In fact, Wernicke (1874) is here speculating about conduction aphasia specifically in deaf and mute persons (“Taubstummen”). In this specific case, tactile and visual rather than acoustic “images” (see Bennett and Hacker 2006 for conceptual problems with this empiricist belief) are the starting point of the psychological reflex arc. Wernicke specifies that the “anatomical location [of these images] is not known sufficiently well, but certainly not identical to the first temporal gyrus [i.e., the location of the acoustic images]”. The anatomical connection between visual or tactile images on the one hand and the corresponding motor images on the other (which Wernicke assumes are located in the same brain area as in people without developmental hearing or speech impairments: Broca’s area), is formed by Burdach’s arched bundle (not quite the AF in the modern sense of the term, as we will discuss below). Interruption of these white matter tracts, Wernicke hypothesizes, leads to conduction aphasia in deaf and mute persons. For people without hearing problems, who have learned to speak in the typical way (i.e., through imitation, as Wernicke argues), Wernicke instead locates the insula as the site which, when lesioned, would lead to conduction aphasia: a_1_, “the central ending of the accoustic nerve” (superior temporal gyrus) is connected to b (inferior frontal gyrus) “through association fibers a_1_ b that run in the insular cortex” (p. 19).

Wernicke did later concede that his view of conduction aphasia being the result of insular lesions was not supported by autopsy findings: There were no cases of patients with conduction aphasia in which the insula was exclusively or predominantly damaged. This concession has since been interpreted by, e.g., Geschwind (1965) to suggest that Wernicke later held the view that the AF was the only tract connecting the two language centers. In fact, Wernicke recognized a ventral as well as a dorsal connection even in his latest, posthumously published writings (Wernicke 1906). Geschwind’s emphasis on Wernicke’s writings in support of the dorsal connection during the early days of the return of neuroanatomy to aphasia research led to the decades-long neglect of the ventral pathway (Weiller et al. 2011).

As mentioned above, what Wernicke called “Burdach’s bundle” should not be equated with the AF in the modern use of the term (i.e., the dorsal fronto-*temporal* tract, (Bullock et al. 2022; Porto et al. 2021)). To be sure, Burdach was the first to refer to the structure as “arcuate fasciculus” (Vavassori et al. 2023) because of its arc-like shape, which is most prominent in the fibers that course into the temporal lobe. However, the figure on page 34, in Wernicke’s trademark style (see Levelt 2013), leaves little doubt that Wernicke is here referring to the part of the dorsal tract which does not project into the temporal lobe: He tentatively places the tactile and visual images c and d in angular gyrus and occipital cortex. (And as is clear from the just cited passage, he is certain that they are not located in the first temporal gyrus.) Like other 19^th^ century authors, such as Arnold (1838), Meynert (1892) and Dejerine, and 20^th^ century authors like Ludwig and Klingler (1956), Wernicke saw the AF as part of a general system of dorsal association fibers. And even today, the terms “arcuate fasciculus” and “superior longitudinal fascicle” (SLF) are by no means used uniformly (Porto de Oliveira et al. 2021).

**Fig. 2:**
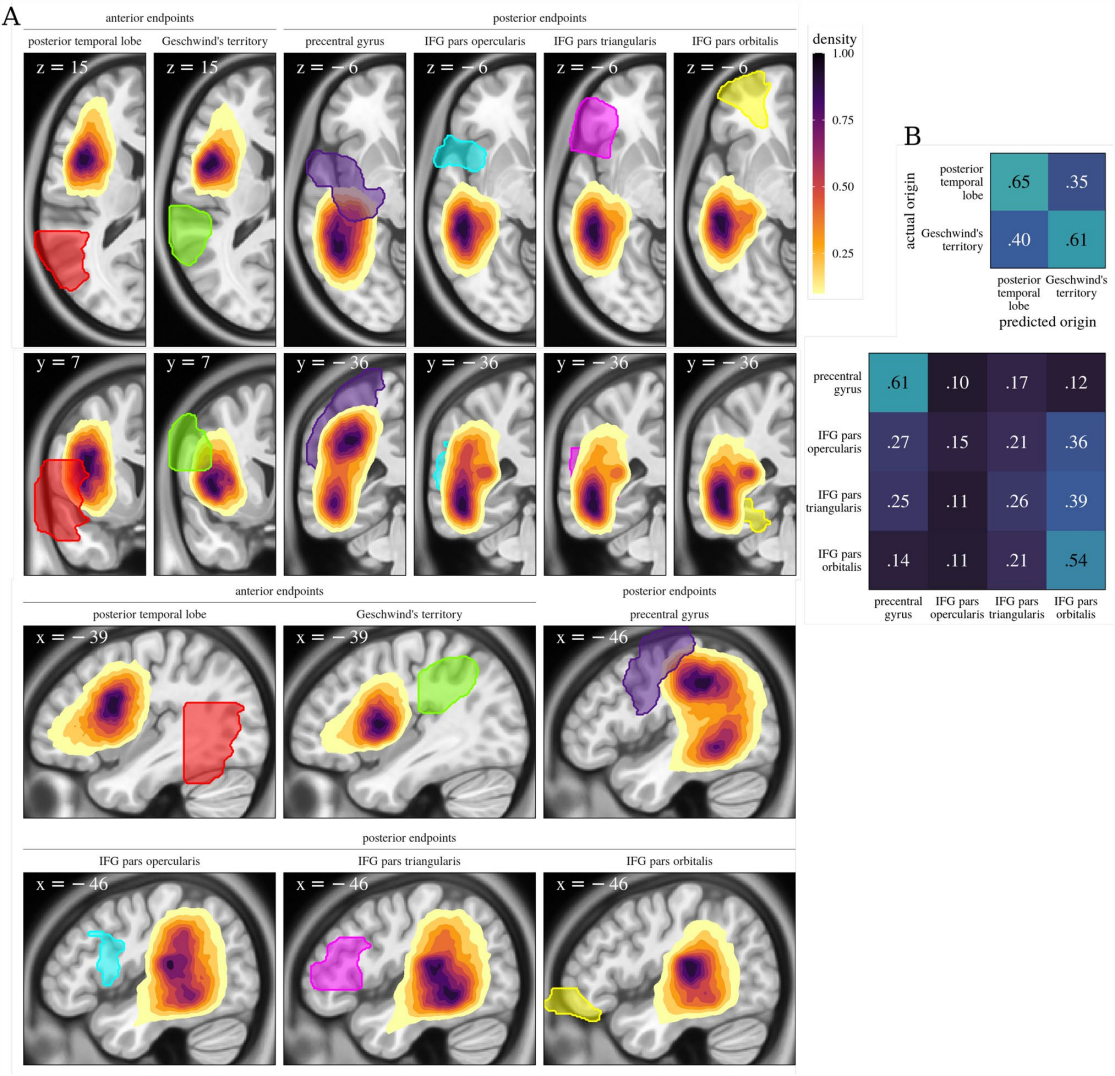
Endpoint distributions of subtracts. **A** Joint marginal endpoint densities of dorsal streamlines in MNI space, per origin ROI (transparent colored silhouettes; all except Geschwind’s territory were taken from the AAL atlas (Tzourio-Mazoyer et al. 2002) and the temporal lobe was truncated such that MNI coordinate $y <= 35$). Background brain slices are those with maximum endpoint density per endpoint location (anterior/posterior). ROI outlines and endpoint density shading are cumulative across all slices, shown and not shown. **B** Confusion matrices of neural network predictions for posterior (top) and anterior (bottom) streamline origin labels

### The alternative conception

In line with this long, recently broken tradition, we believe that both the direct AF subtract and the anterior indirect subtract (as well as other possible subdivisions of the AF (Janssen et al. 2022)) are better conceived as part of a continuous dorsal association tract system, analogous to the continuous ventral association tract system (Weiller et al. 2021). The reason is that anatomically, it is difficult to differentiate between the tracts. The subtracts in question have been laid bare through dissection (Fernández-Miranda et al. 2008; Martino et al. 2013) and can reliably be found using DTI tractography—it is not doubted that they exist in some sense of the word “exist”. However, the relevant question is in which sense they exist: like a fossil hidden inside a rock or rather like the David in the marble before Michaelangelo carved it out.

In white matter bottlenecks, streamlines intermingle and their endpoint distributions are not easily distinguishable. We can illustrate this using global tractography (Reisert et al. 2011) on participants from the Human Connectime Project (HCP, Van Essen et al. 2013). We defined dorsal subtracts (i.e., bundles of streamlines traversing a lateral dorsal bottleneck below the central sulcus in an anterior-posterior orientation (Weiller et al. 2022)) on the basis of their cortical endpoints on only one of the sides of the dorsal bottleneck along the anterior-posterior axis. (Here, we are still ignoring many of the U-shaped association fibers which briefly join the stream only to part from it after the first sulcus they encounter.) Subsequently, we inspected their end point distributions on the other side. Figures 1 and 2 show that whether the first endpoint is in Geschwind’s area or posterior temporal lobe matters very little with regard to the distribution of anterior endpoints. A three-layer neural network with layers of 12, 6 and 3 hidden units trained on 200k streamlines from 181 participants from the HCP’s young adult dataset (https://www.humanconnectome.org/study/hcp-young-adult/document/1200-subjects-data-release) could correctly classify only 63% of unseen posterior endpoint labels (“Geschwind’s area” or “posterior temporal lobe”) based on anterior endpoint coordinates (c.f. the chance level of 50%). Conversely, if the dorsal subtracts are defined by their frontal endpoints (IFG pars triangularis, orbitalis or opercularis, or PreCG), their posterior endpoint distributions also largely overlap: Accuracy of the corresponding neural network was 39%, where the improvement from baseline (25%) can largely be attributed to relatively accurate discrimination between streamlines stemming from PreCG and those stemming from pars orbitalis, the part of IFG distal to PreCG—the former being more richly connected to the parietal lobe than the latter.

Doubtless, these results depend on the global tracking methodology to a degree. So-called “walker-based” tractography (used by Catani et al. 2005) has a stronger tendency than global tractography to retain the topology of the set of streamlines, which results in higher mutual predictability between the two sets of endpoint coordinates for the former method compared to the latter (and smoother-looking tracts). It is an unsettled question which of these models is a closer approximation to reality, but it should be noted that within non-human primates’ fiber bundles, single axons intertwine even over short distances (Rockland 2018). Other studies, including histological assesments (Leergaard et al. 2010; Ronen et al. 2014; Lee et al. 2019) and advanced diffusion measurements (Dhital et al. 2019; Leergaard et al. 2010; Ronen et al. 2014; Veraart et al. 2019; Kunz et al. 2018) also hint at highly dispersed and intermingling fibers (angular deviations of about 20–30$^{\circ }$ on the millimeter scale) even in the most coherent regions. Taken together, these studies suggest that global tractography delivers the more realistic results. The more white matter fibers truly intermingle, the weaker the case is for assigning a label to discrete subtracts.

Of course, one can always define subtracts by means of their cortical endpoints. As Meynert (1892) already noted (with some hyperbole), “the association systems probably connect different cortical areas ($\ldots$) so abundantly that no two ($\ldots$) cortical areas remain physiologically unconnected”. But the reasons for doing this would have to be functional rather than anatomical. The merit of dividing the dorsal stream into a subtract ending above the Sylvian fissure and a subtract ending below it will depend primarily on whether such a division elucidates or rather obfuscates our understanding of the behavior of patients with damage to the fibers in these tracts. (And even then the question remains what is gained by endowing the subtract thus found with a name rather than describing it as, for instance, “dorsal white matter fibers connecting A and B while traversing C”—naming the tract risks reifying it.) Such evaluation may be done, for instance, through model comparison, where variance in behavior explained by atrophy of—or lesions to—the dorsal system as a whole is compared to variance explained by the loss of integrity of its presumed subtracts. As it stands, the anatomical partitions are taken as given. That, we believe, is premature.

## Supplementary Information

Below is the link to the electronic supplementary material.

Supplementary file1 (gz 3 KB)

Supplementary file2 (gz 2 KB)

Supplementary file3 (txt 2 KB)

## Data Availability

Supplementary materials include the relevant Human Connectome Project participant IDs as well as ROIs that are not already publicly available or straightforward to create using information supplied in the manuscript.
